# A long-distance translocation initiated an outbreak of raccoon rabies in Hamilton, Ontario, Canada

**DOI:** 10.1371/journal.pntd.0008113

**Published:** 2020-03-25

**Authors:** Susan Nadin-Davis, Tore Buchanan, Larissa Nituch, Christine Fehlner-Gardiner

**Affiliations:** 1 National Reference Laboratory for Rabies, Ottawa Laboratory–Fallowfield, Canadian Food Inspection Agency, Ottawa, Ontario, Canada; 2 Wildlife Research and Monitoring Section, Ontario Ministry of Natural Resources and Forestry, Trent University, Peterborough, Ontario, Canada; Universitetet i Oslo, NORWAY

## Abstract

Despite proactive measures to prevent raccoon rabies entering Canada from the United States, several incursions of this disease have occurred. The largest outbreak, first reported in December 2015 in the city of Hamilton, Ontario, has resulted in the reporting of 449 animal cases as of December 31, 2018. Initial phylogenetic studies on the index case suggested that this outbreak was not due to local cross-border spread from the Niagara region of the United States where raccoon rabies has persisted for several years. Phylogenetic analysis of whole genome sequences of a viral collection from the Hamilton area and several US states indicates that a long-distance translocation of a diseased animal from southeastern New York State was responsible for this incursion. The role of the skunk as a potential secondary host supporting persistence and / or spread of the virus is also examined.

## Author summary

Distinct variants of rabies virus are harbored by several wildlife species across North America. The variant associated with the raccoon host is especially problematic given its rapid spread throughout the eastern seaboard of the United States over the second half of the 20^th^ century and the high population density of this species in urban habitats that raises the potential for disease spread to both companion animals and their owners. Accordingly, extensive efforts to control raccoon rabies have been undertaken at many jurisdictional levels. This study uses current methods of genome analysis to explore the origins of a raccoon rabies outbreak in and around the city of Hamilton, Ontario, Canada, located close to the border with western New York state. In contrast to previous Canadian outbreaks of raccoon rabies, which were due to cross-border spread of the virus, this study demonstrates that the incursion into the Hamilton area was the result of a long-distance animal translocation from south-eastern New York. The implications of this event for strategies to control raccoon rabies are discussed.

## Introduction

Rabies virus is an important zoonotic pathogen, distinct variants of which are maintained by populations of canid and meso-canid host species worldwide [1]. In North America several viral variants are associated exclusively with wildlife species, and one such variant, the raccoon rabies virus (RRV), first recognized in Florida in the 1940s, is now widely disseminated throughout the eastern seaboard of the United States [2]. The raccoon (*Procyon lotor*) is a highly adaptable species which thrives in urban environments and as a consequence raccoon rabies is a serious concern for both public and animal health. Accordingly, the province of Ontario has engaged in proactive vaccination campaigns along areas of the US-Canada border to prevent incursions of raccoon rabies into eastern Ontario. These measures include immunizing raccoons along selected border areas of eastern Ontario and the Niagara peninsula in efforts to maintain population immunity [3]. Despite these measures, since 1999 outbreaks of raccoon rabies have occurred in Ontario [4–6] and in the eastern Canadian provinces of New Brunswick [6] and Quebec [7]. The number of reported cases associated with each of these incursions has varied between one, due to a single case in Quebec in 2015, and over 100 for outbreaks in Ontario (1999–2005) and Quebec (2006–2009) [5, 8]. In response to most of these situations costly control programs and additional surveillance activities were implemented to eliminate these cross-border incursions from US states. Currently an outbreak in Hamilton, Ontario, which began in 2015, has recorded 449 animal cases as of December 31, 2018 and is thus by far the largest incursion of RRV into Canada to date. The presence of raccoon rabies in this highly urban area was of particular concern and a wide variety of activities at the provincial and municipal levels were initiated to eliminate the disease and limit its public health impact [9].

Improved knowledge of factors that impact disease spread would be extremely helpful in informing efforts to minimize the incidence and severity of future outbreaks. Previous molecular epidemiological methods lacked the resolution to explore RRV spread in detail [10, 11] but current highly parallel sequencing technologies that facilitate entire rabies virus genome characterization can effectively track viral evolution and spread [5] [12]. A prior study that included the index case from the Hamilton outbreak suggested that, unlike previous RRV incursions into Canada which were all the result of natural cross-border animal movements, this outbreak was probably not due to viral spread from the region of New York state in close proximity to the city of Hamilton [8]. This study seeks to define the likely origins of the Hamilton RRV variant by an extensive comparison of this virus with variants circulating across eastern North America. The resulting analysis provides a detailed picture of the complex pattern of RRV variants circulating in New York State and also explores the potential role of the skunk host in disease maintenance and spread during the Hamilton outbreak.

## Materials and methods

### Samples and sequencing

All Canadian RRV samples were diagnosed by the direct fluorescent antibody test (FAT) applied to brain smears [13] by the National Reference Laboratory for Rabies located in Nepean, Ontario. In many instances the samples had originally been collected as part of an enhanced surveillance activity in support of a raccoon rabies control operation and diagnosed as positive using the direct rapid immunohistochemical test (DRIT) [14]; these samples were subsequently submitted to the National Reference Laboratory for Rabies for confirmatory testing using the FAT. Antigenic typing performed as described [15] confirmed the presence of the raccoon variant. Brain material from these cases was maintained in long-term storage at -80°C.

Between the start of the Hamilton outbreak in 2015 and mid-June 2017, raccoons comprised 68.3% of all diagnosed cases, skunks made up 30.5% and other species the remaining 1.2% [9]. This study employed a subset (n = 84, S1 Table) of all laboratory confirmed cases (n = 338) reflecting this time-frame and host species representation with 58 raccoons (69.0%), 25 skunks (29.8%) and one red fox (1.2%). Sample selection was also representative of the geographical distribution of the outbreak. The study also drew extensively on previously generated whole genome sequencing (WGS) data for samples from other Canadian provinces and US states [5, 8, 12], in particular a large collection of samples from New York state, collected between the early 1990s to the present, some of which are described here for the first time (see S2 Table).

Total RNA extraction from brain tissue, its use as template for whole RRV genome amplification and viral sequencing by Illumina technology were performed as previously described [5]. All sequence files generated during the course of this work are available through the NCBI database (Accession numbers MK540658-540886).

### Phylogeographic analysis

MEGA v7 [16] was used for RRV sequence alignments, genetic distance computations, selection of the best fitting nucleotide substitution model of the RRV dataset and for generation of Maximum Likelihood (ML) trees. Phylogenies were generated using datasets in which terminal PCR target regions were removed to eliminate any bias these might introduce. Coalescent analysis was performed by two independent runs of 50 million Markov chain Monte Carlo (MCMC) iterations, with 10% burn-in, with BEAST v1.8.2 [17] and the BEAGLE library [18], using the GTR+G model and a relaxed molecular clock. Duplicate runs were checked for convergence using Tracer (v1.7), all trees were combined using Logcombiner (v1.8.4) and the optimal time-scaled maximum clade credibility (MCC) tree was produced with Tree Annotator (v1.8.4) and illustrated using Figtree (v1.4.3).

### Evaluation of protein variation between viral clades

Complete genome sequences of the 84 Hamilton outbreak samples were edited with MEGA v7 to generate separate alignments for each of the five viral genes for translation to protein sequences. Differences from the index case were scored manually.

### Map generation

The township from which each sample originated was used to determine the latitude and longitude co-ordinates employed for mapping of viral variants using ArcView GIS v10.1 software.

## Results

### Source of the Hamilton RRV variant

**Fig 1:**
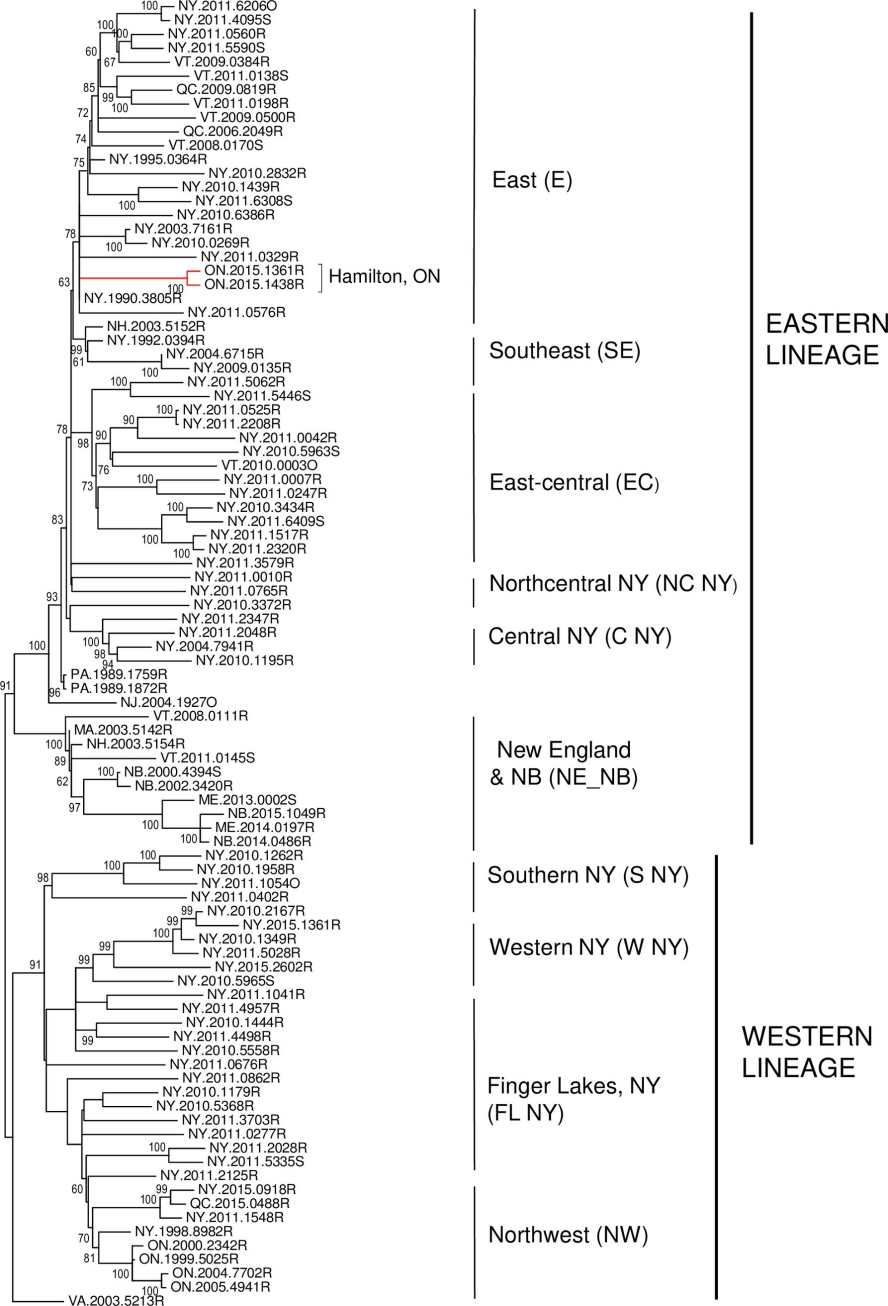
Phylogenetic comparison of two Hamilton RRV samples with isolates from across Canada and the northeastern USA. A dataset of 11861 positions for 94 samples was employed in a ML analysis using the GTR+G model and 500 bootstrap replicates. The tree is drawn to scale with branch lengths representing number of substitutions per site according to the scale at bottom and bootstrap values > 50% are indicated at all major nodes. The Hamilton samples are identified (red branch) and the main viral groupings are indicated to the right of the tree.

**Fig 2:**
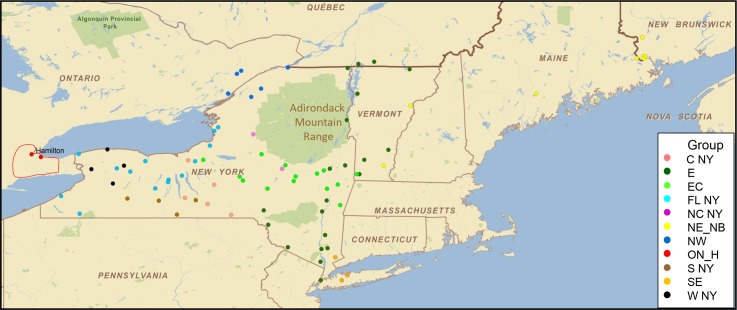
Distribution of the RRV variants across the northeastern USA and Canada border areas. The geographical locations of most samples analyzed in the phylogeny presented in Fig 1, including the position of the city of Hamilton, are shown. The extent of the outbreak area around the city as of June 2018 is indicated by a solid red line. Viral variants, as identified in Fig 1, are color-coded as indicated in the legend; ON_H identifies Hamilton RRV samples.

Whole genome sequencing was completed for 84 samples representative of the raccoon rabies outbreak in Hamilton, Ontario. In a preliminary analysis, two of these samples, both collected in 2015, were compared to RRV samples from across northeastern North America including isolates from previous Canadian outbreaks, several New England states and Pennsylvania and an extensive collection from New York State. The resulting phylogeny of 94 RRV samples (Fig 1) identifies two main clades, designated as eastern and western RRV lineages, and a single outlier from Virginia. As annotated in Fig 1 and illustrated in Fig 2, these clades are further subdivided into groups that exhibit varying degrees of spatial localisation. Within the eastern lineage six distinct groups are identified. The large East group (E) is widely distributed throughout eastern New York State and includes isolates recovered previously from Vermont and Quebec. Notably this group includes the two Hamilton samples which cluster amongst samples originating from eastern New York. The eastern lineage also includes a southeast (SE) group of four samples recovered from the extreme southeastern area of New York and New Hampshire, a large East-central (EC) group spread across much of central and eastern parts of New York, two distinct isolates recovered from Northcentral New York (NC NY) and a central New York (C NY) group. Early samples from Pennsylvania and one from New Jersey form outliers to these five groups while samples from the New England states and the province of New Brunswick (NE_NB) form a separate outlying group within the eastern lineage. The western lineage is subdivided into four viral groups thus: the southern New York (S NY) group; a large collection recovered from the Finger Lakes region (FL NY) that extends into western New York and from which the Northwest (NW) group, responsible for previous incursions into eastern Ontario, emerged; and a western (W NY) group recovered exclusively from counties close to the Niagara peninsula. The western and Finger Lakes viral types have both been recovered from the western zone that abuts the US-Canada border located about 85 km from the city of Hamilton. However, these viruses are all phylogenetically very distinct from the Hamilton variant thus confirming earlier suggestions that, unlike all previous incursions of RRV into Canada, the Hamilton outbreak was not caused by localised cross-border spread. Moreover, the virus responsible for this most recent incursion was unrelated to previous Canadian isolates.

The clear east-west dichotomy of the New York samples has been described previously [10, 11] and its emergence was further investigated during this study using historical samples collected in the 1990s (S1 Fig). These early samples supported the occurrence of two separate incursions of RRV into New York, in accord with surveillance records of the time [19], and these progenitor viruses subsequently emerged into the groups currently circulating throughout the state. The samples recovered from the southeastern part of the state, which were closely related to four samples collected in Pennsylvania in 1989, gave rise to the eastern lineage groups (E, SE, EC NC & C) while those recovered from counties in the southwest evolved to yield the western lineage groups (S, W, FL & NW) as identified in Figs 1 and 2. While some mixing of the S, C and FL NY viruses is apparent within the southcentral finger lakes region, the poor raccoon habitat of this mountainous and well forested area, which includes several large lakes, appears to have limited east-west virus spread. Similarly, the Adirondack Mountains in the northern part of the state have acted as a barrier to disease incursion.

**Fig 3:**
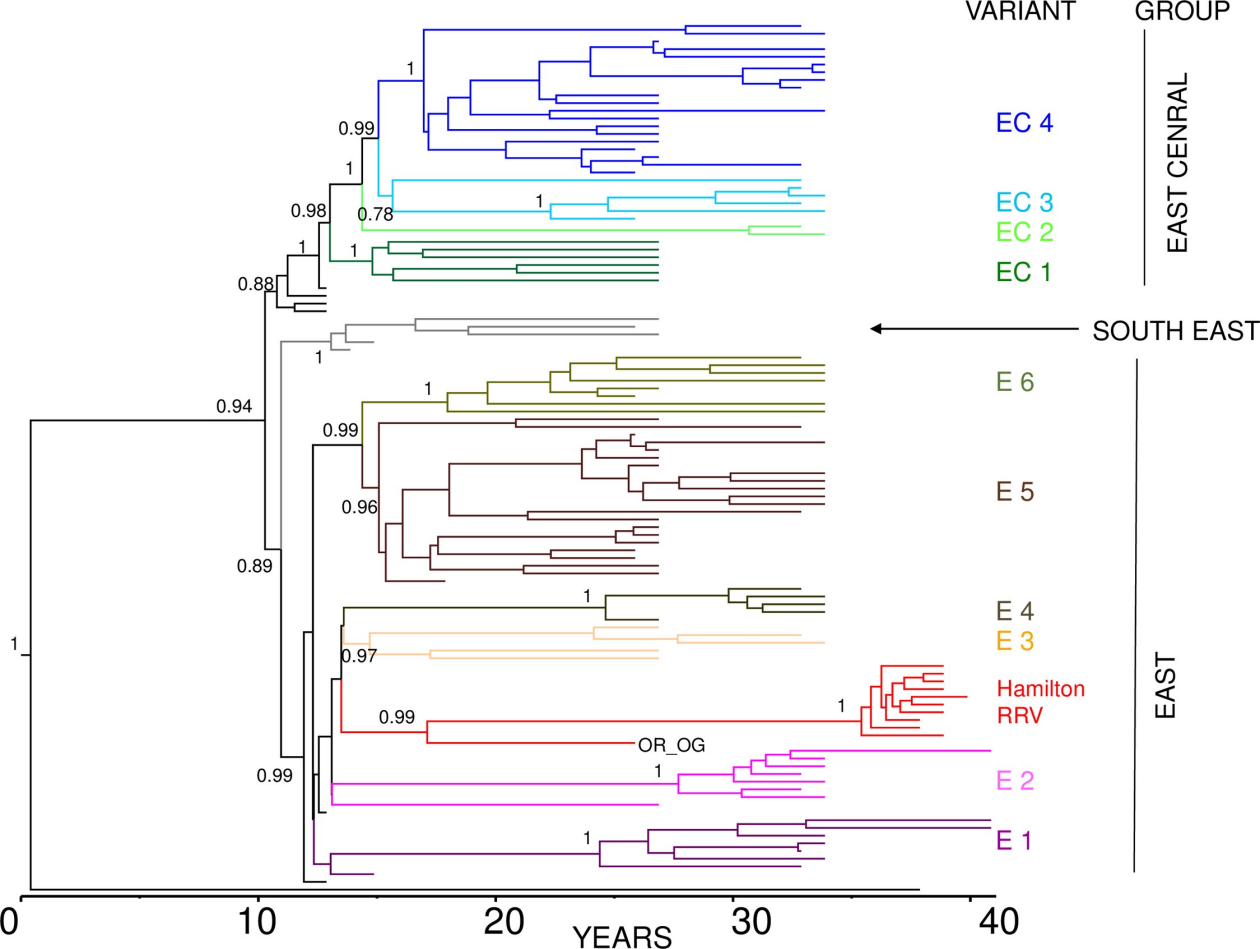
Phylogenetic comparison of representative Hamilton RRV samples with isolates from counties in eastern New York. This MCC tree was generated by coalescent analysis performed on 112 samples, including 10 representatives of the Hamilton outbreak, and a member of the NY W group included as an outgroup. Color-coded variants are identified to the right of the tree; early samples from the 1990s which do not cluster with any confidence to a specific clade remain in black. The sample clustering as an outlier to the Hamilton RRV group (NY.2003.7427) is represented as OR_OG. Posterior values for many major branch points are shown to the left of the node. The scale at bottom indicates the timeframe in years.

**Fig 4:**
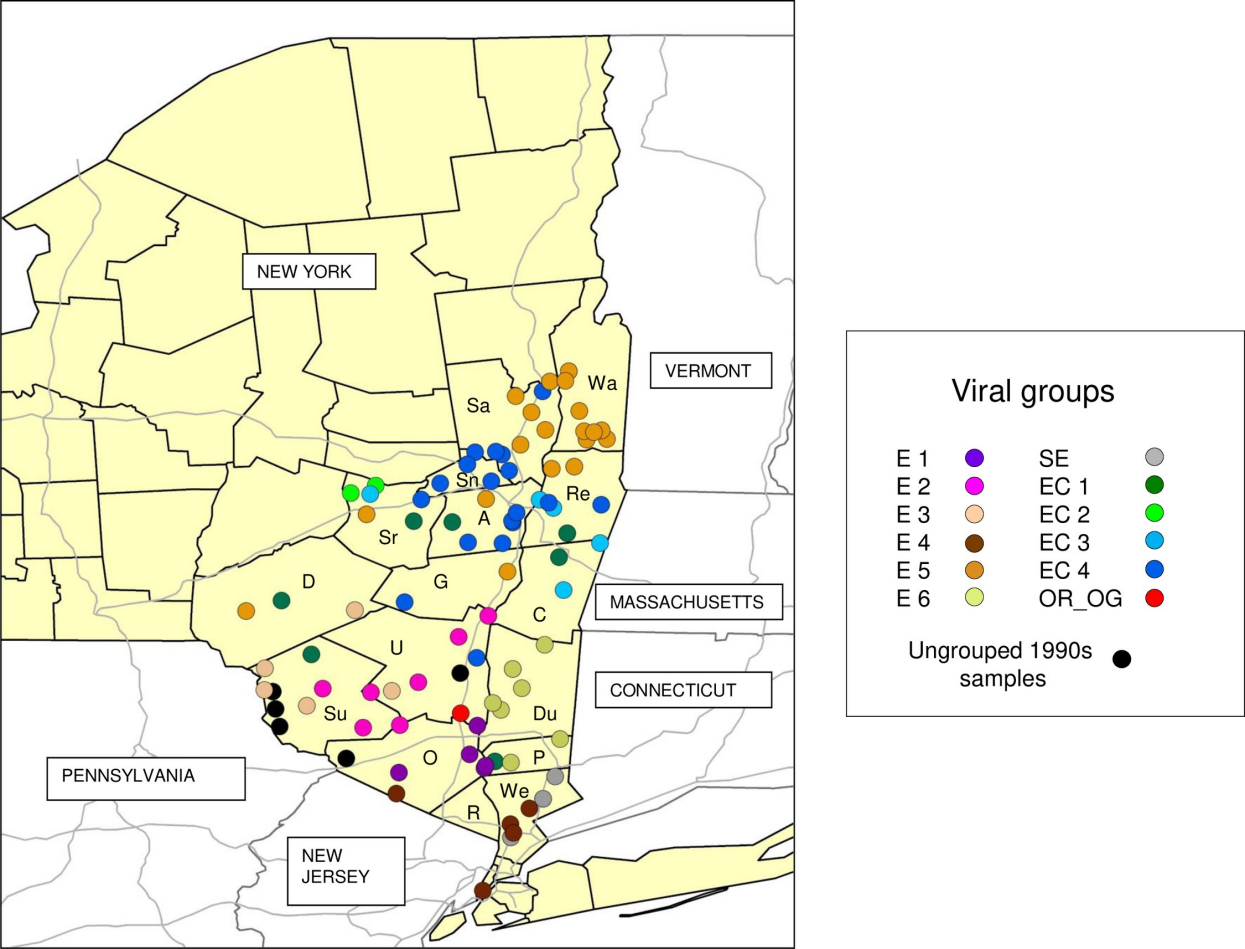
Distribution of the RRV variants of southeastern New York. Isolates are color-coded as shown in the legend and as per Fig 3. The New York counties in the target region are shown in beige with black boundaries and are identified as follows: those in the Capital District include Albany (A), Columbia (C), Greene (G), Rensselaer (Re), Saratoga, (Sa), Schenectady (Sn), and Washington (Wa); those in the Hudson Valley which runs north-south and separates the easternmost counties of Dutchess (Du), Putnam (P) and Westchester (We) from western counties Orange (O), Rockland (R), Sullivan (Su) and Ulster (U). The counties of Delaware (D) and Schoharie (Sr) are located further west in neighboring zones. Main highways are shown in grey and bordering states are identified.

To try to identify the origin of the Hamilton outbreak more precisely, a detailed time-scaled phylogenetic analysis was performed using 10 samples representative of the diversity of the Hamilton RRV outbreak and additional samples comprising additional members of the E, EC and SE viral groups from eastern New York, the region identified as harboring viruses most closely related to those from Hamilton (Fig 3). While the SE variant was again represented by a very small number of genetically closely related samples, the other two groups were further subdivided into 10 distinct variants: E 1 to E 6 and EC 1 to EC 4 that have emerged over the last 29 years since introduction of RRV into eastern NY in 1990. As illustrated in Fig 4 and summarised in S2 Table, these viral variants exhibit various degrees of spatial localisation. For example, E 1 and E 2 were found only west of the Hudson valley in Orange, Sullivan and Ulster counties while E 3 was also found further west in Delaware county and E 4 had spread into Westchester county east of the Hudson river and south into New York city. A single sample (NY.2003.7427) from Ulster County that formed a separate branch (OR_OG) within this section of the tree was the only sample that clustered strongly as an outlier to the monophyletic clade formed by the 10 Hamilton samples. These data show that the Hamilton outbreak was due to the introduction of a single viral variant related to viruses of the EAST group. The age of the Hamilton clade was estimated at 5.5 years (95% HPD 4.4–6.7 years) suggesting that the incursion occurred around 2013, several months prior to its recognition in 2015.

Members of the SE group were recovered from just two counties, Westchester and Putnam, in the most southeastern part of the state consistent with prior observations (Figs 1 and 2). Viral variants of the EC group (EC 1 to EC 4) appear to have various spatial ranges; EC 2 was recovered from a single county only, while others (EC 1 and EC 4) were more widely distributed in several counties west of the Hudson valley.

### Phylogeny of the Hamilton RRV variant–potential role of the skunk as host

**Fig 5:**
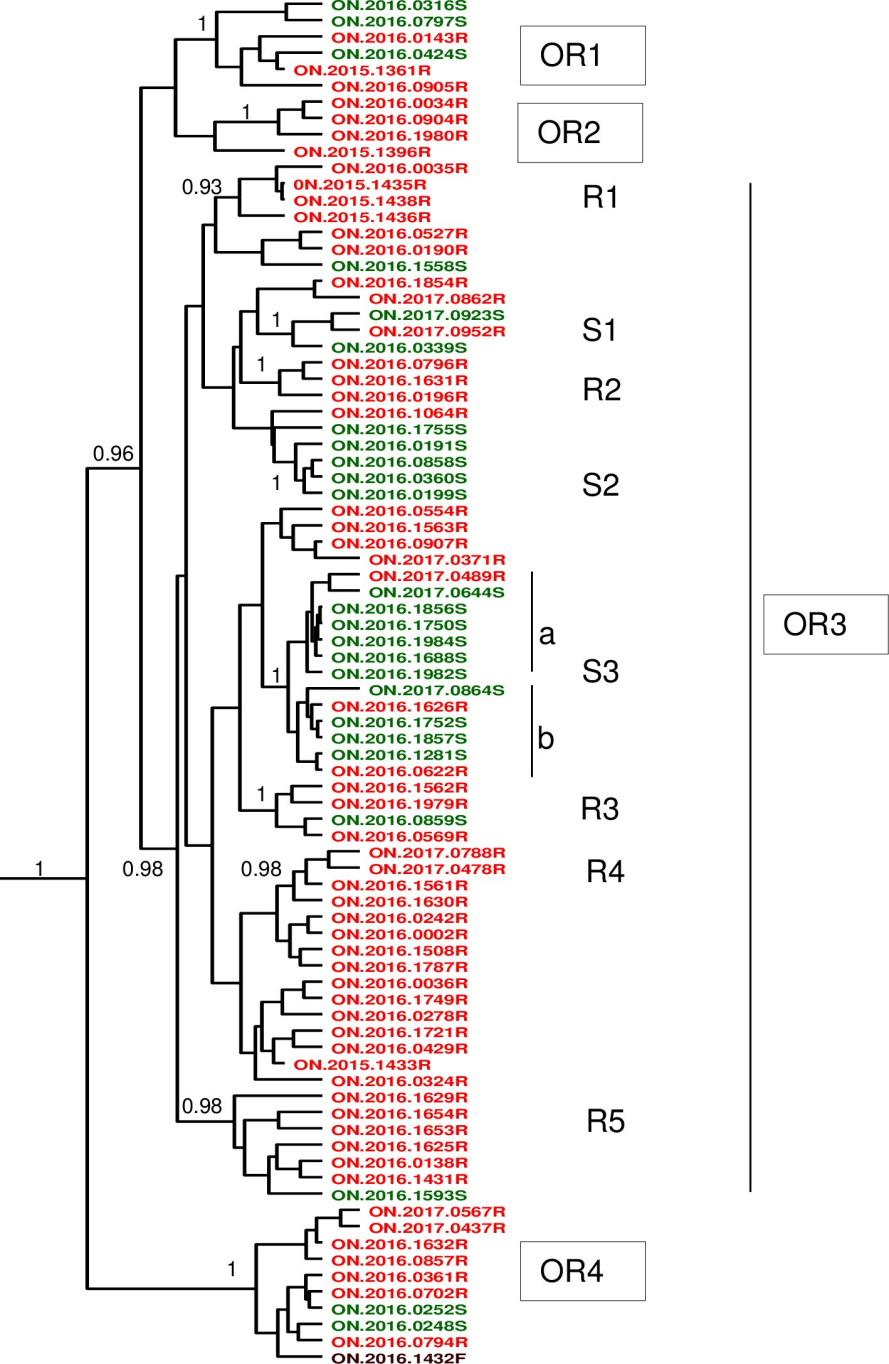
Phylogeny of the Hamilton RRV outbreak. An MCC tree of 84 samples was generated by coalescent analysis. The host species for each sample is indicated by the final letter in the sample name and by its color coding as follows: F, fox (brown); R, raccoon (red); S, skunk (green). Clades are identified to the right of the tree. Posterior values ≥ 0.7 are shown to the left of the nodes. Sample NY.2003.7427R was used as outgroup.

The recent emergence of this RRV outbreak provided an opportunity to explore the evolution of this RRV variant in real-time. An MCC tree, generated using all 84 sequenced RRV samples from Hamilton and the related NY.2003.7427R isolate as outgroup, identified four well-supported Ontario raccoon (OR) clades (OR1 to OR4) with posterior values of ≥ 0.96 (Fig 5). OR4, represented by 10 samples, is the earliest offshoot from the main clade. The largest clade, OR3, contains a number of samples which do not exhibit any further clustering with any confidence (posterior values < 0.7) but it also includes a number of smaller well-supported clades designated R1 to R5 and S1 to S3 according to the predominant host species (R, raccoon or S, skunk). The latter three clades comprise a total of 20 samples of which only four came from raccoons with the rest recovered from skunks thus: S1, 2 of 3 samples (66.7%); S2, 4 of 4 samples, with another skunk sample as outlier (100%); S3, 10 of 13 samples (76.9%). This observation prompted exploration of a potential role of skunks in viral maintenance. The distribution of all 84 samples according to host species clearly shows clustering of most skunk-associated samples in the western and northwestern part of the study area from which samples contained within clades S1 –S3 were recovered (S2A and S2B Fig). Indeed, samples that clustered together phylogenetically generally clustered together spatially as well (see groups OR3-R3, R4, R5) as would be expected for localised spread of variants as they emerged.

To explore whether host association correlated with certain coding changes within the virus, the five open reading frames of all 84 samples were compared. Using the index case (ON.2015.1361R) as reference, a total of just 39 amino acid substitutions were identified within the dataset and these were distributed among all five genes thus: N, 1; P, 5; M, 3; G, 11; L, 19. Out of these 39 substitutions, 27 represented changes in a single isolate only and another seven substitutions occurred in just two isolates that were not closely related; these changes probably reflect neutral mutational drift and are unlikely to be functionally significant. The nature of the remaining five substitutions, which occurred in specific phylogenetic groups, is summarised in Table 1.

**Table 1:** Characteristics of five amino acid substitutions identified within the Hamilton RRV sample set.

Group	Gene	Amino Acid position	Change compared to reference sequence
OR3_S2	P	48	Ser to Asn
OR4	L	49	Arg to Lys
OR3_R1 except for ON.2015.1436	L	607	Leu to Ser
OR3_R1	L	1087	Arg to Lys
OR3_S3a	L	1139	Gly to Arg

## Discussion

This study, has explored the origins of the Hamilton RRV outbreak, using highly detailed phylogenetic reconstruction possible through the availability of a large collection of whole genome sequences of RRV specimens collected from a number of jurisdictions [5, 8, 12]. It should be noted that the extent of this outbreak has been recognised as a result of a large-scale enhanced surveillance program initiated by the province of Ontario in which sick and found-dead animals of rabies vector species were collected and tested for rabies. This activity yielded much larger numbers of cases than would have been reported through the regular passive surveillance system currently in operation throughout Canada.

This study has confirmed prior suggestions that the virus responsible for the Hamilton outbreak did not spread from the adjacent border area of western New York [8] but originated from an entirely different area of the state. Specifically, the data suggest that southeastern New York was the source of this virus (Figs 1 and 2). One sample recovered in 2003 (NY.2003.7427R) that originated from the town of Plattekill in Ulster County clustered with strong support with the Hamilton samples (Figs 3 and 4). However, given that the NY.2003.7427R and Hamilton samples were collected at least 12 years apart a direct connection between them cannot be inferred but the data do indicate that the Hamilton samples cluster with strong support (posterior value of 0.99) within the EAST New York group, and in particular with variants E 1 to E 4 which appear to be restricted in their distribution to southeastern counties bordering the Hudson Valley as well as the neighbouring counties of Delaware and Schoharie. The evidence strongly supports this area as being the source of the Hamilton RRV variant, almost certainly through translocation of an infected animal given the distances involved.

Precedence for RRV spread as a result of long distance movement of raccoons by anthropomorphic activities include the emergence of the mid-Atlantic raccoon rabies variant in Virginia in the 1970s [20] while hitch-hiking of infected animals on board long distance transport trucks has also been identified as a likely means of inadvertent disease spread [21]. A network of US interstate highways and a Canadian highway which connect southeastern New York with the city of Hamilton are extensively used for trade between the two nations and could readily provide routes by which a diseased stowaway raccoon might travel between the two areas. Alternatively, but less likely, there is the possibility of transfer by ship travelling between the ports of New York and Hamilton.

Compared to previous incursions of RRV into Canada in which the raccoon was the primary, and sometimes the only, species affected, the Hamilton outbreak is notable in that about 30% of all laboratory confirmed cases were reported in skunks. As the skunk is a well-recognised reservoir for other rabies virus variants [22] and is clearly susceptible to infection with RRV, this host could potentially play a role in disease spread. Clustering of cases recorded predominantly in skunks infected with specific variants (S1, S2, and S3 of the OR3 clade) in the northwestern part of the study area was noted. To explore the possibility that independent emergence of skunk-associated variants might be occurring, changes in the nature of the viral proteins associated with these cases were explored.

Compared to the sequence of the reference index case (ON.2015.1361R), most nucleotide changes in all 84 sequenced samples were either synonymous in nature or non-synonymous in just one or two samples and thus unlikely to have contributed to any viral adaptation. Furthermore a small number of changes identified within specific groups of viruses (Table 1) appear unlikely to be functionally significant when compared to sequences of other North American skunk-associated lineages [23].

Indeed, it is likely that these are neutral changes that became fixed in these viruses during emergence of these groups.

Both skunks and raccoons occur at relatively high densities in urban environments so significant rates of inter-species interaction are likely; accordingly, skunks could provide a source of virus for infection of raccoons and thus play some role in viral spread. Notably a study of raccoon rabies in the state of Vermont could not rule out limited local skunk to skunk virus transmission but it provided no evidence that skunks play a role in long term maintenance of the virus [12]. Furthermore, the possibility of bias in the Hamilton study due to higher levels of collection of rabid skunks compared to raccoons during active surveillance cannot be ruled out.

The identification of the source of the Hamilton outbreak as a long-distance translocation has implications for wildlife rabies control strategies. Maintenance of an immune population of raccoons along the Niagara border area of Canada effectively prevented rabies from crossing into Ontario between 1994 and 2013. However, this strategy was ineffective in preventing the Hamilton incursion in which anthropomorphic activity appears to have transported the infected animal through the control zone into an area where the raccoon population remained naïve to rabies infection. Prevention of a recurrence of such events will involve different strategies in which both border officials and personnel involved in long distance transportation of goods are made fully aware of the disease hazards of stowaway wildlife.

This outbreak also had a profound impact on public health services in the region. An extensive education and awareness campaign concerning the dangers posed by this outbreak to both humans and domestic animals was undertaken; furthermore, the administration of rabies postexposure prophylaxis to potentially exposed people increased by 52% during the first year of the outbreak [9]. Mitigation of the large social and financial costs of such an event is best achieved, as in this situation, by networking of all relevant stakeholders and preparative planning of One Health strategies to address all aspects of the public health response and disease control.

## Supporting information

S1 Fig Phylogeny of early NY and PA RRV samples. (TIF)

S2 Fig Spatial distribution of 84 sequenced Hamilton RRV samples based on viral variant (A) or host species (B).
(TIF)

S1 Table List of samples from the Hamilton raccoon rabies outbreak included in study. (XLSX)

S2 Table RRV samples used for comparative genomics with the Hamilton RRV group. (XLSX)
