# Supplementary figures and images for: A long-distance translocation initiated an outbreak of raccoon rabies in Hamilton, Ontario, Canada
Source: PLoS Negl Trop Dis. 2020 Mar 25;14(3):e0008113. doi: 10.1371/journal.pntd.0008113 (PMC7135350; doi:10.1371/journal.pntd.0008113)

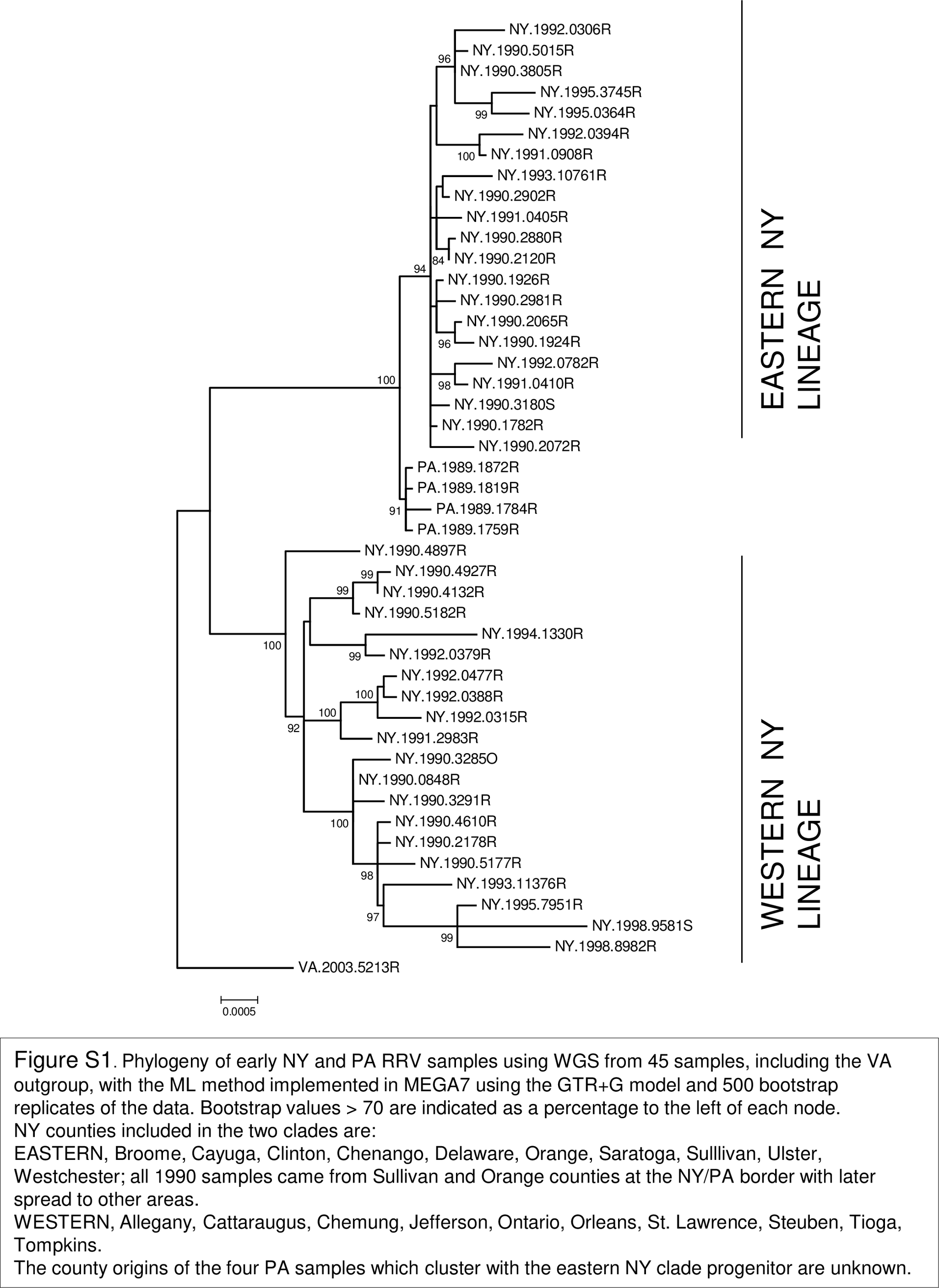

Supplement: S1 Fig — (TIF) [file pntd.0008113.s001.tif]

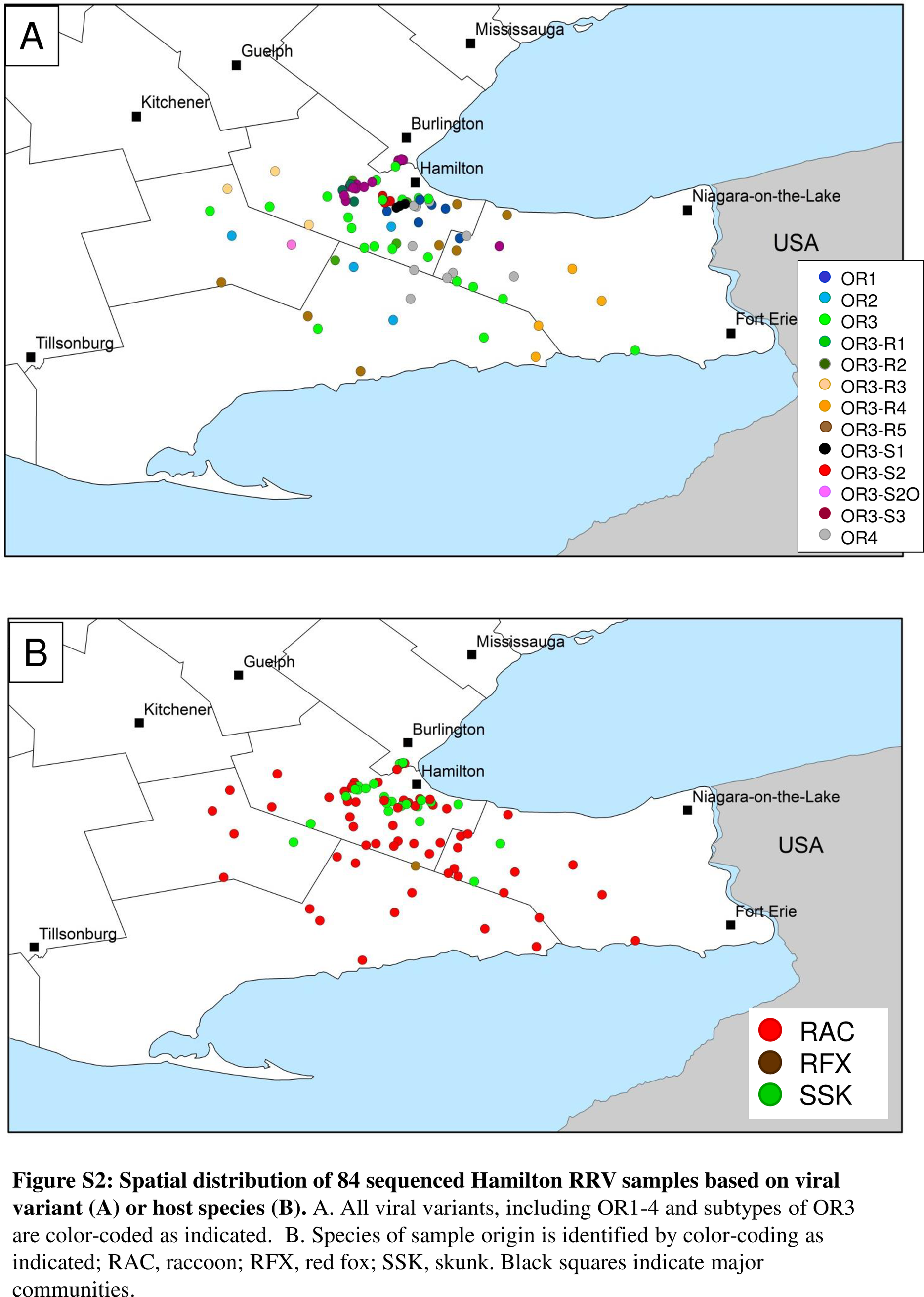

Supplement: S2 Fig — Spatial distribution of 84 sequenced Hamilton RRV samples based on viral variant (A) or host species (B). (TIF) [file pntd.0008113.s002.tif]
